# Role of C-Reactive Protein in Diabetic Inflammation

**DOI:** 10.1155/2022/3706508

**Published:** 2022-05-17

**Authors:** Julijana Stanimirovic, Jelena Radovanovic, Katarina Banjac, Milan Obradovic, Magbubah Essack, Sonja Zafirovic, Zoran Gluvic, Takashi Gojobori, Esma R. Isenovic

**Affiliations:** ^1^Department of Radiobiology and Molecular Genetics, VINČA Institute of Nuclear Sciences-National Institute of the Republic of Serbia, University of Belgrade, Belgrade, Serbia; ^2^King Abdullah University of Science and Technology (KAUST), Computer, Electrical, and Mathematical Sciences and Engineering (CEMSE) Division, Computational Bioscience Research Center (CBRC), Thuwal, Saudi Arabia; ^3^University Clinical-Hospital Centre Zemun-Belgrade, Clinic of Internal Medicine, School of Medicine, University of Belgrade, Belgrade, Serbia

## Abstract

Even though type 2 diabetes mellitus (T2DM) represents a worldwide chronic health issue that affects about 462 million people, specific underlying determinants of insulin resistance (IR) and impaired insulin secretion are still unknown. There is growing evidence that chronic subclinical inflammation is a triggering factor in the origin of T2DM. Increased C-reactive protein (CRP) levels have been linked to excess body weight since adipocytes produce tumor necrosis factor *α* (TNF-*α*) and interleukin 6 (IL-6), which are pivotal factors for CRP stimulation. Furthermore, it is known that hepatocytes produce relatively low rates of CRP in physiological conditions compared to T2DM patients, in which elevated levels of inflammatory markers are reported, including CRP. CRP also participates in endothelial dysfunction, the production of vasodilators, and vascular remodeling, and increased CRP level is closely associated with vascular system pathology and metabolic syndrome. In addition, insulin-based therapies may alter CRP levels in T2DM. Therefore, determining and clarifying the underlying CRP mechanism of T2DM is imperative for novel preventive and diagnostic procedures. Overall, CRP is one of the possible targets for T2DM progression and understanding the connection between insulin and inflammation may be helpful in clinical treatment and prevention approaches.

## 1. Introduction

Type 2 diabetes mellitus (T2DM) is becoming a prime global health problem. The prevalence of T2DM quadrupled during the past 35 years and still has a constant growth projection [1, 2]. Moreover, we anticipate that T2DM incidence would dramatically increase with increased adolescent obesity [3, 4].

Diabetes is a long-term metabolic disease in which the impaired ability of the body produce and/or respond to the hormone insulin. T2DM is characterized by abnormally elevated blood glucose levels, which affect the kidneys, heart, and blood vessels. Most diagnosed diabetes cases are type 1 diabetes mellitus (T1DM) or T2DM. T1DM, also known as juvenile diabetes, is characterized by an absolute insulin deficiency. On the other hand, T2DM is a progressive disease sustained by insulin resistance (IR) and beta cell dysfunction [5]. The most common way to diagnose diabetes in patients is by measuring fasting plasma glucose (FPG). FPG levels less than 100 mg/dL (5.6 mmol/L) are normal glucose levels in the blood, while levels ranging from 100 to 125 mg/dL (5.6 to 6.9 mmol/L) are considered a prediabetes indicator. Patients are only diagnosed with diabetes after at least two separate tests show FPG levels higher than 126 mg/dL (7 mmol/L). Hemoglobin A1C (HbA1C) is also an indicator of T2DM, which indicates the quality of diabetes treatment since it provides healthcare professionals with information about the average blood glucose levels in the past two or three months.

Accumulating evidence corroborates the crucial role of inflammation in T2DM pathologies [1, 6]. Low-grade inflammation characterized by elevated inflammatory protein levels, including C-reactive protein (CRP), is linked with T2DM pathogenesis [1, 7–9]. CRP, the typical inflammatory biomarker produced in the liver, is regulated by adipocyte-derived proinflammatory cytokines, including interleukin 6 (IL-6) and tumor necrosis factor *α* (TNF-*α*) [10, 11]. The level of CRP is usually low in healthy individuals but can elevate 100- to 200-fold or higher in acute systemic inflammation [12] and is chronically elevated in patients with T2DM. In individuals with T2DM, CRP levels range between 4.49 and 16.48 mg/L [13, 14] and among individuals with acute systemic inflammatory response syndromes from 31.08 [15] to 226.1 mg/L [16].

The production of CRP may be triggered by many metabolic and inflammatory factors associated with the development of T2DM, such as increased blood glucose, adipokines, and free fatty acid levels. In addition, an increased level of CRP represents a reliable predictor of vascular complications and progression of cardiovascular disease in diabetic patients [17, 18]. Furthermore, numerous human [1, 19, 20] and animal studies [21–23] demonstrated the associations of elevated serum CRP levels with obesity and the progression of IR leading to T2DM. These findings add to the notion that the inflammatory state demonstrated by higher CRP levels is an essential factor in the pathogenesis of T2DM. Numerous studies report a significant positive association between elevated CRP levels and the risk of T2DM development [1, 7–9, 24]. On the other hand, some studies lack this association after adjusting for many factors contributing to T2DM, including adiposity and hyperinsulinemia [20, 25]. Since increased body fat and obesity are among the main factors in the development of T2DM, which are also associated with increased risk for progression of obesity-related IR and inflammation, we review the published literature to collate and provide a comprehensive summary of the relationship between T2DM and CRP.

## 2. CRP

**Figure 1:**
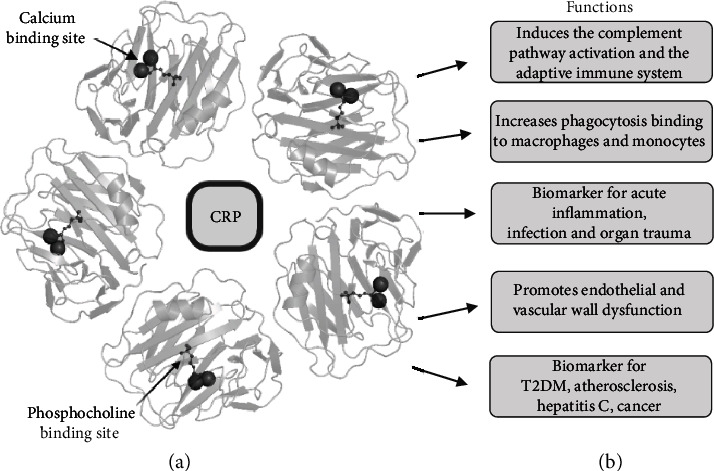
(a) C-reactive protein (CRP) crystal pentametric structure with calcium and phosphocholine binding sites. (b) Functions of CRP. CRP: C-reactive protein. Biorender.com and Protein Data Bank in Europe were used to generate the ribbon diagram of the CRP complex.

CRP was first described as a serum protein capable of precipitating C-polysaccharide pneumococcal cell walls during the acute phase of infective conditions in the presence of calcium [26–28]. We know CRP belongs to a conserved protein family called pentraxins and has been identified in several organisms ranging from arthropods to humans [29, 30]. Structurally, it is a 206 amino acid cyclic pentameric protein with five identical subunits noncovalently connected, with a molecular weight of ~23 kDa (Figure 1) [31]. Each of the five subunits is similar to a discoid orientation toward a central pore folded into two antiparallel two-layered *β*-sheets [32]. Native CRP (nCRP) dissociates to monomeric/modified isoform of CRP (mCRP) across lysophosphatidylcholine in platelets, apoptotic monocytic THP-1, and Jurkat T cells [33, 34]. Moreover, a study by Ji et al. demonstrates that nCRP, when bound to the cell membrane, dissociates into subunits while retaining some native conformation before entirely dissociating into mCRP subunits, which detaches from the membrane [35]. This intermediate isoform, termed mCRP_m_, seems to have similar biological functions as mCRP, including enhancing the classical complement pathway activation and promoting proinflammatory activity [36]. Unlike these two isoforms, nCRP displays more anti-inflammatory activities, probably because it limits the production of the membrane attack complex (MAC) and C5a, thus inhibiting the alternative complement activation [37]. mCRP has strong angiogenic effects, both *in vitro* and *in vivo*, and likely leads to the neovascularization of tissues in which it is deposited or synthesized [38]. Thus, treating CRP-mediated pathological conditions could include a novel therapeutic strategy that inhibits mCRP activity [39] or prevents the dissociation of nCRP into mCRP.

CRP can recognize and bind to endogenous damage-associated molecular patterns (DAMPs) and exogenous pathogen-associated molecular patterns (PAMPs). Thus, CRP initiates an immune response and contributes to eliminating various pathogens and damaged necrotic or apoptotic cells [40, 41]. After binding to a specific ligand, CRP manifests anti-inflammatory features by activating the C1q molecule in the classic complement pathway engaging C3, the main adhesion molecule of the complement system, and the terminal membrane attack complex, C5–C9. In this way, CRP leads to the opsonization of the pathogen [42, 43]. However, CRP also acts as a proinflammatory mediator that binds to the Fc*γ* receptors of IgG, leading to the release of proinflammatory cytokines [11, 44, 45].

### 2.1. CRP Levels and Detection

The Pentraxin 1 (PTX1) gene encodes CRP, and in humans, it is located on chromosome 1q21–q23 on the long arm and consists of a long 3′ untranslated region and 2 exons [46]. The first exon encodes two amino acids and a signal peptide, and the second exon encodes 204 amino acids [47]. Human CRP binds to phosphocholine (PCh) across five PCh-binding locations, and every individual subunit links two calcium ions [29]. Bacterial, eukaryotic, fungal, and endothelial cells induce PCh, and its binding to CRP is mediated mainly through Phe^66^ and Glu^81^ [32, 48, 49]. The C1q molecule binds and stimulates classical complement pathways on the CRP contrary site, particularly C1, C4, and C2 [43, 47]. Baseline CRP levels are affected substantially by polymorphisms in the noncoding regions in the promoter and the untranslated region. Polymorphisms of IL-6 and IL-1 genes, which stimulate CRP production, also affect the baseline CRP levels [50]. Groups of CRP single-nucleotide polymorphisms (SNPs) inherited together are known as five common main haplotypes, of which two are associated with higher baseline CRP levels, and two are associated with lower baseline CRP levels [51]. Moreover, these haplotypes influence the acute phase level of CRP and the development of several diseases [50, 52].

CRP participates in acute phase response to inflammation, infection, or organ trauma in humans, increasing up to 1000-fold within 24 to 72 hours [53, 54]. CRP is found in several cell types, including neurons, epithelial cells, monocytes, lymphocytes, and smooth muscle cells. However, the CRP gene is primarily induced in the hepatocytes due to elevated inflammatory cytokines, dominantly IL-6 [46]. Still, these extrahepatic sites do not impact CRP levels in plasma [47]. The CRP half-life is approximately 19 hours, and its average levels are generally presented as mg/L or mg/dL [55, 56]. Nonetheless, CRP levels differ among laboratories since there is no optimized standard. Levels below 0.3 mg/dL are considered physiological, while levels above 10 mg/dL indicate bacterial and virus infections and severe tissue damage [57].

Several methods are in use for CRP detection and measurement. Immunoturbidimetry is the most commonly used method for clinical CRP determination [58, 59]. Conventional enzyme-linked immunosorbent assay (ELISA) and fluorescence-linked immunosorbent assay (FLISA) have also been widely used for the quantification of CRP [33, 60]. The main limitations of these methods are the use of complex features and the lack of cost-effectiveness. Recently, CdSe/ZnS quantum dot-based FLISA and Histag-HRP functionalized nanoconjugate-based immunoassays were developed [61, 62]. The main advantages of these two assays are high sensitivity, reduced time for analysis, and expanded detection range for serum samples. In addition, the assays provide a lower detection limit (LOD) and, consequently, facilitate early detection of the CRP biomarker, which is imperative for prompt diagnosis. Novel electrochemical biosensor platforms and microfluxgate sensor systems contribute to these requirements [63–65].

Levels of CRP are directly correlated with the presence and elimination of inflammatory agents [57]. CRP's role as a diagnostic and prognostic biomarker has been established for acute infections [66–69], as well as for various chronic conditions, such as T2DM [1], atherosclerosis [70, 71], hepatitis C [72], and different types of cancer [73, 74]. Accumulating evidence suggests a connection between the activation of the complement system and the pathogenesis of T2DM [75–77].

## 3. T2DM and Inflammation

**Figure 2:**
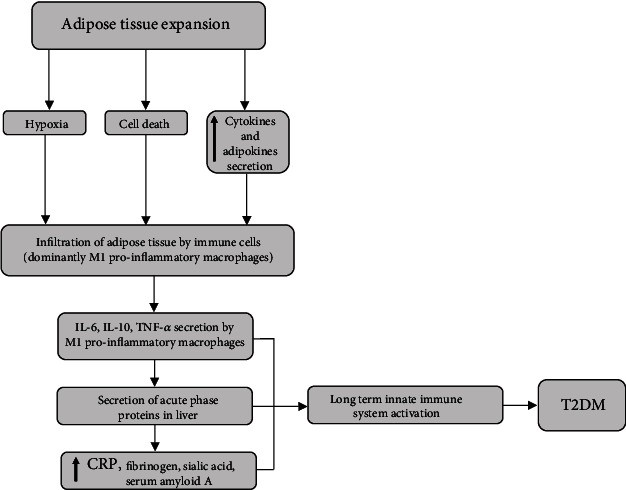
T2DM development as a result of chronic low-grade inflammation. CRP: C-reactive protein; IL-6: interleukin 6; IL-10: interleukin 10; i.p.: intraperitoneal; TNF-*α*: tumor necrosis factor *α*; T2DM: type 2 diabetes mellitus.

T2DM is a systemic, noncommunicable disease with multiple metabolic disorders, characterized by defects in insulin secretion and/or insulin action leading to hyperglycemia [78]. Chronic hyperglycemia induces oxidative stress, inflammation, and local and whole-body failures [79]. Of all the types of diabetes, two forms are most common: T1DM and T2DM. T1DM occurs due to autoimmune destruction of a critical mass of pancreatic *β* cells, which causes a lack of insulin synthesis and secretion [80]. Therefore, T1DM is an autoimmune disease with the rate of *β* cell destruction varying from rapid to slow descent [80, 81]. T1DM patients are rarely obese and often develop other autoimmune disorders such as Hashimoto's thyroiditis, Graves' disease, and vitiligo [79]. This suggests that multiple genetic and environmental factors may induce the autoimmune destruction of *β* cells. Contrarily, T2DM is the most prevalent form, with a frequency of 90-95% of all cases in the population. T2DM characterizes a whole spectrum of events, from insulin secretion defects to the impaired action of different enzymes regulated by insulin [79, 82]. There are numerous causes of T2DM, but unlike T1DM, autoimmune destruction of *β* cells never occurs [79]. The disease at earlier stages frequently goes undiagnosed because classic symptoms develop gradually as hyperglycemia develops, and insulin levels in such patients are often normal or elevated [83]. Age, obesity, and lack of physical activity are probably the most crucial factors affecting the development of T2DM [79, 84]. Long before the clinical manifestation of T2DM, IR occurs, characterized by hyperinsulinemia often combined with obesity, hypertension, and dyslipidemia [85]. During IR, compensatory hyperinsulinemia helps maintain normal glucose levels [83], but *β* cells lose their ability to overcome IR through hypersecretion, which leads to hyperglycemia [83, 85]. Subclinical inflammation is an essential part of IR, and various inflammatory markers correlate with IR, including CRP [82, 86, 87]. The long-term activation of the innate immune system results in the development and progression of T2DM instead of reestablishing the normal physiological state (Figure 2). Besides CRP, the innate immune system produces acute phase response proteins, fibrinogen, and serum amyloid A, whose levels noticeably change in response to infection, tissue injury, or inflammation [88]. High levels of CRP, fibrinogen, sialic acid, serum amyloid A, and low albumin and transferrin levels have been linked with T2DM occurrence [89].

Adipose tissue, especially visceral white adipose tissue (WAT), plays a significant role in T2DM's inflammatory process and development. In addition, studies demonstrated that human adipocytes could produce CRP under the stimulation of several proinflammatory cytokines, suggesting a link between obesity and its comorbidities, including IR [90–92]. CRP mRNA levels in human adipose tissue also positively correlated with IL-6 mRNA levels. CRP expression *in vitro* was also increased by IL-6 and lipopolysaccharide stimulation, contributing to the elevated plasma CRP levels found in obese individuals [93]. Moreover, human CRP overexpression in transgenic mice fed a high-fat diet contributed to the development of IR, hepatosteatosis, adiponectin downregulation, and expression of proinflammatory cytokines in epididymal adipose tissue [94]. These data indicate the role of CRP in the pathogenesis of obesity-induced metabolic disorders [94]. A recent study reported a significant decrease in weight gain and food intake, and improved insulin sensitivity was observed in CRP knockout rats placed on a high-fat diet. These results suggest that CRP is not only a biomarker of inflammation but also has a crucial role in energy balance, body weight, insulin sensitivity, and glucose homeostasis [95]. In addition, numerous molecules such as cytokines and other bioactive substances involved in the inflammatory pathways are produced and secreted by WAT [96]. The abdominally distributed WAT plays an essential role in the inflammatory process [79].

Furthermore, macrophages and immune cells infiltrate as the adipose tissue expands, contributing to local and whole-body low-grade inflammation. Adipose tissue expansion does not follow simultaneous capillaries' development, which causes adipocytes to become too distant from the vasculature [97]. Thus, hypoxia, adipocyte cell death, and increased secretion of chemokines and adipokines may be part of the mechanisms that initiate adipose tissue inflammation [98]. Infiltration of adipose tissue is accompanied by a decrease in anti-inflammatory M2-type macrophages, while the number of proinflammatory M1-type macrophages increases [96]. Although T2DM does not imply autoimmune destruction of *β* cells, the inflammatory process in the pancreas islets does occur. It is still unclear what induces that process, but it appears to be highly dependent on IL-1 action [96].

Adipocyte-derived bioactive metabolites like leptin and adiponectin are also involved in T2DM pathogenesis [96]. The leptin levels in serum are directly proportional to the total fat mass; its production increases during inflammation. Also, leptin activates and modulates innate and adaptive immune responses and promotes proinflammatory pathways [96, 99]. Contrarily to leptin, adiponectin is involved in anti-inflammatory pathways. Thus, low adiponectin levels are associated with T2DM incidence, which shows an inverse relationship between plasma adiponectin levels and CRP levels [95, 100]. Additionally, TNF-*α*, IL-6, and IL-10 are essential cytokines produced by adipocytes and immune cells. TNF-*α* and IL-6 are known proinflammatory factors, whereas IL-10 exhibits anti-inflammatory properties [96], and IL-6 predominantly controls CRP production by hepatocytes [86]. Besides IL-6 and TNF-*α*, the liver produces IL-1, which stimulates the synthesis of acute-phase proteins [89]. Also, the correlation between fasting insulin and CRP levels implicates the link between IR and inflammatory processes [78, 101, 102].

Inflammation of adipose tissue and high production of TNF-*α*, IL-6, and IL-1*β* in obesity is vital for T2DM incidence and progression [103]. Some authors indicated that TNF-*α* contributes to the development of IR in skeletal muscle of human individuals by suppressing Akt substrate [104] and stimulating IL-18 expression [105]. Furthermore, TNF-*α* constrains insulin-stimulated glucose uptake and endothelium vasodilation [106]. In addition, TNF-*α* receptors mediate the upregulation of the NF-*κ*B pathway and influence proteins that disturb insulin signaling and proinflammatory response [107]. Nevertheless, IL-6 affects insulin-degrading enzyme expression and activity in the liver and skeletal muscle tissues, and modulation of this enzyme may contribute to T2DM and obesity [108]. According to a new meta-analysis, IL-6 indeed mediates chronic inflammation in T2DM. However, IL-6 influence on the general population seems insufficient, and acting on the IL-6 pathway may not reduce the risk for T2DM occurrence [109]. Even though IL-6 is essential for liver homeostasis, only a few cell types express IL-6 receptors, including hepatocytes, and its persistent activation is associated with liver pathologies [110]. Studies demonstrate that IL-6-inducible protein SOSC-3 promotes IR by direct binding to insulin receptors and inhibiting its kinase activity [111]. However, the results of one study indicate anti-inflammatory properties and the homeostatic role of IL-6 in obesity-associated inflammation and IR [112]. Also, it was demonstrated that IL-6 increases the responsiveness of macrophages to IL-4 and thus balances its shifting toward proinflammatory M1 macrophages [112]. In addition, the results from many studies also confirmed the pleiotropic nature of IL-6 and indicate that the effects of IL-6 on inflammation differs depending on the duration of exposure, tissue type, and factors such as concentration and source of IL-6 [113–117].

Growing evidence indicates the role of gut microbiota in the immune system regulation and pathogenesis of T2DM [118–120]. The product of certain bacteria triggers an inflammatory cascade, including recruitment of interleukins and CRP [121, 122], which leads to impaired insulin action and T2DM development [123, 124]. The level of imidazole propionate (ImP), a microbial-produced histidine metabolite, is higher in subjects with T2DM [125]. Furthermore, the proposed mechanism by which microbial-derived ImP diminished glucose metabolism includes activating the p38*γ*-mTOR1-S6K1 signaling cascade that caused insulin receptor substrate degradation and inflammation [125, 126]. In addition, increased circulating levels of LPS have been recognized as an important marker that implies a link between variations in microbiota composition and inflammation in T2DM [122]. LPS promotes IR via stimulation of Toll-like receptors on adipocytes, upregulation of NFkB, and activation of cytokines TNF-*α* and IL-6 [127–129]. In addition, lower diversity in the gut microbiota is associated with increased white blood cell counts and high sensitivity CRP (hs-CRP) levels [121, 130].

Some evidence indicates the role of CRP in diabetes-induced microvascular complications, such as neuropathy, retinopathy, and nephropathy. Elevated glucose levels could trigger microvascular alterations and increased production of inflammatory factors, including CRP, IL-6, and TNF-*α* [131]. Serum hs-CRP is linked with diabetic neuropathy occurrence, one of the most prevalent diabetes complications [132–134]. Nevertheless, in male and female patients, symptomatic peripheral diabetic neuropathy and inflammation are related to endothelial dysfunction and elevated CRP serum levels [135]. It is assumed that in patients with T2DM peripheral neuropathy, increased CRP levels are positively correlated with inflammation grade [133]. Furthermore, according to data from a large cohort study where participants were monitored for one year, hs-CRP levels above 2.5 mg/L could predict neuropathy complications in T2DM [136]. Considering hs-CRP low-cost and accessibility features, it might be a useful predictable biomarker for neurovascular disorder in T2DM [131].

Diabetic retinopathy (DR) is the leading cause of visual loss worldwide. Several studies have investigated the relationship between CRP levels and DR with inconsistent results. Qiu et al. observed a positive correlation between elevated hs-CRP levels in the blood and DR onset and progression [137]. In contrast, Song et al. reported that CRP levels could be associated only with the severity of DR [138]. Recently, it was shown that CRP levels could be associated with the stage of DR in T2DM patients [139]. However, previous studies demonstrated that patients with higher CRP and BMI levels were less likely to develop DR [140]. Inconsistent results from various studies may be caused by ethnical differences in CRP and BMI levels and excluding some clinical parameters such as duration of DM and complex genetic and environmental variations.

Diabetic nephropathy (DN) is a leading cause of end-stage renal disease. Numerous studies confirmed higher CRP levels in T2DM patients with DN [141–145]. A study by Tang et al. demonstrated the pathogenic role of CRP in renal inflammation and fibrosis using diabetic animal models [141]. The meta-analysis also confirmed higher hs-CRP levels in T2DM patients with DN than in healthy subjects and T2DM patients without DN [142]. Hayashino et al. reported that serum hs-CRP levels could be a helpful factor for predicting the risk of DN developing in T2DM patients [143]. Additionally, it was shown that higher hs-CRP levels were associated with DN complications in T2DM patients [145].

Although these findings open new doors in understanding diabetes pathology, further research is needed to answer current ambiguities.

## 4. T2DM and CRP

### 4.1. Evidence from Animal Studies

Numerous animal studies have shown the essential role of CRP in infections and inflammatory processes (Table 1). CRP is synthesized in response to monocytic mediators such as IL-1 and IL-6 in the acute phase of infections [11]. CRP recognizes and binds to specific polysaccharides in the bacterial wall and induces further complement pathway activation, leading to the opsonization of pathogens [146]. Moreover, there is evidence of CRP involvement in proliferation and apoptotic processes through activation of Fc receptors and the consequent production of proinflammatory mediators and proapoptotic cytokines [147]. In addition, growing evidence show CRP is not only an inflammatory marker as its level is proven to be elevated in T2DM cases (Table 1).

Zou et al. showed elevated serum CRP levels in male streptozotocin-induced Sprague-Dawley rats compared to untreated rats [148]. The same authors noticed degenerative changes in the heart of streptozotocin-induced rats, such as irregular cardiac muscle fibers and degenerative necrosis [148]. Furthermore, Shirpoor et al. investigated the effect of vitamin E on oxidized low-density lipoprotein, lipid profile, CRP, and VSMC proliferation of rat aorta in streptozotocin-induced Wistar rats. They found elevated CRP levels in the diabetic rats compared to the untreated rats [21]. Additionally, the same authors reported that the antioxidative effects of vitamin E attenuated the level of CRP and arterial complications in diabetic rats [21]. Using the same model, Talebi-Garakani and Safarzade showed that four weeks of resistance training decreases serum inflammatory markers in diabetic rats, including CRP levels [22]. These authors also reported increased CRP levels in the diabetic rats compared to the untreated ones.

We have previously shown that rats with high-fat diet-induced obesity and IR develop cardiac hypertrophy and elevated CRP levels in serum [23]. We also showed that estradiol treatment leads to CRP level reduction and significantly reduces heart mass in treated rats compared to untreated obese rats [23]. In another study, Cho and colleagues reported the differentially expressed proteins in the kidney, eye, aorta, and serum of diabetic rats compared to the controls [149]. They reported elevated serum CRP levels in diabetic rats compared to untreated rats. Beyond the high CRP level, they studied protein alterations in the vasculature and found three potential biomarkers in aorta lysate samples that could present early signs of diabetic vascular complications development [149]. The Asgary et al. study also indicated CRP increases in alloxan-induced diabetic rats compared to untreated. The same authors concluded that four weeks of pumpkin powder administration decreased glucose, cholesterol, triglyceride, LDL, and CRP levels in diabetic rats compared to untreated diabetic rats [150]. Interestingly, Ige et al. reported similar CRP values, and also, histological pancreas findings were entirely physiological, with small amounts of secretions in diabetic and control group rats [151].

The variance in study design may explain the differences in CRP levels in diabetic animals. Although alloxan induces diabetes through a different mechanism (oxidative stress through Fenton reaction) than streptozotocin (alkylation of DNA) [152], higher doses of alloxan applied to male and female rats induce the same effects on diabetes development and alterations of CRP levels in circulation, as streptozotocin [150, 153, 154]. Some authors suggest that infections in T2DM should be treated with antibiotics and indicate that medication with fluoroquinolone antibiotics may decrease elevated CRP values in diabetic rats [155]. Nevertheless, Almatroodi et al. showed that Thymoquinone, the key active component of the medicinal plant *Nigella sativa,* improved CRP, antioxidant enzymes, liver enzymes, and inflammatory markers levels in diabetic rats [156].

### 4.2. Evidence from Human Studies

Accumulating evidence from human-based studies suggests that inflammation contributes to the pathogenesis of T2DM (Table 2). Several human studies show elevated CRP levels correlate with the development of T2DM, even without adjustment of other parameters, such as adiposity, hyperinsulinemia, hypertriglyceridemia, and low HDL cholesterol [1, 7–9, 24].

The West of Scotland Coronary Prevention Study showed that elevated CRP indicates T2DM development in men (middle-aged), independent of established risk factors, such as fasting plasma triglyceride, body mass index (BMI), and glucose [10]. Pradhan et al. [19] also observed elevated CRP levels in diabetic middle-aged women compared to the healthy control, supporting a possible role of inflammation in the pathogenesis of T2DM. Furthermore, Han et al. reported sex differences in the association of elevated CRP levels with the incidence of T2DM [9]. This strong association in women may be explained by the hormone differences and the higher adiposity percentage [157]. A study by Nakanishi et al. [158] showed the influence of CRP on T2DM development in Japanese Americans and not in the original Japanese population, which probably indicates the impact of different lifestyles on T2DM manifestation. The possible explanation considering the link between CRP and T2DM development may be the role of oxidative stress in inducing hyperglycemia [159], which further promotes inflammatory response and elevation of CRP [160]. Furthermore, it was found that oxidative stress might impair insulin endocytosis in endothelial cells [161] and thus could lead to endothelial dysfunction and IR [158, 162]. Doi et al. [163] reported a clear link between elevated CRP levels and obesity-induced hyperglycemia and T2DM in relatively lean Asian populations (Japanese population) in both sexes even after adjustment for comprehensive risk factors related to IR. Also, in the study by Marques-Vidal et al. [164], higher hs-CRP levels were associated with all T2DM and IR markers, and these associations persevered after multivariate adjustment. Similarly, the study showed that participants with impaired glucose tolerance had higher hs-CRP levels than euglycemic subjects, although this difference became nonsignificant after BMI adjustment. Likewise, Akbarzadeh et al. [165] reported a significant positive association between the hs-CRP level and IR markers (HOMA-IR and FIRI) and a negative association between hs-CRP level and insulin sensitivity markers (QUICKI, McAuley. and Bennett indexes). Some studies showed an association between elevated levels of CRP and leptin in diabetic, obese, and CVD subjects [166–169], suggesting the possible role of CRP in modulating leptin action [170]. In addition, variations in leptin levels were independently associated with CRP [171], indicating that a regulatory loop interrelates CRP and leptin levels. Furthermore, exploring the mechanisms of leptin resistance adds to the notion that CRP, particularly the smaller mCRP, may change the action of leptin by binding to the extracellular domain of the leptin receptor [170]. In this way, CRP contributes to the pathogenesis of obesity-related diseases, including T2DM and CVD [166–169, 172]. A study by Kanmani et al. [1] showed a positive association between CRP levels and the incidence of T2DM in a large Korean population. In addition, the association was more noticeable among the older group (≥50 years), and the combination of CRP levels with obesity and hypertension led to increased incidence of T2DM. Lainampetch et al. [173] reported that patients with increased baseline levels of CRP were at increased risk of developing T2DM. These findings support the premise that CRP may indirectly impair insulin sensitivity and production due to increased systemic inflammation through innate immune response alteration [174]. Also, elevated CRP levels influence the production of adhesion molecules, including E-selectin, intercellular adhesion molecule-1 (ICAM-1), and vascular cell adhesion molecule-1 (VCAM-1) that are directly involved in the regulation of insulin action and local IR [175]. A recent retrospective case-control study [176] imparted that elevated hs-CRP and FPG, insulin, HbA1c, HOMA-IR, and IL-6 were found in T2DM patients. The same authors concluded that obesity-induced dyslipidemia (also demonstrated in the study) causes IR and the subsequent increase in levels of inflammatory markers [176]. Chronic inflammation in obesity disrupts glucose homeostasis causing a persistent increase in blood glucose levels [87, 177]. Also, IL-6 produced by the adipose tissues may stimulate CRP secretion [178], which further increases IR and initiates low-grade inflammation leading to the development of T2DM [176]. On the other hand, some studies suggest that higher CRP levels and T2DM development are attenuated or missing after adjustment to a wide range of confounding factors, including adiposity and insulin sensitivity [20, 25]. The degree of adiposity and baseline glycemia adjustment could explain the heterogeneity between studies and suggests that CRP might not be an independent risk factor for T2DM development [6].

Regarding testing CRP levels, there is a standard CRP assay measuring lower (baseline) levels of this biomarker, contributing to the diagnosis of acute inflammation. Also, a frequently used and a more sensitive hs-CRP assay reflects low-grade chronic inflammatory processes having a predictive value of future CVD risk [179]. In addition, emerging evidence supports the use of hs-CRP levels for CVD risk assessment in IR-diabetic and nondiabetic subjects [180, 181]. The limitation of the abovementioned human studies, which used CRP and not hs-CRP, might be apparent. However, treatment with pioglitazone, which has insulin-sensitizing and anti-inflammatory properties (combined with antilipidemic statin therapy) decreases hs-CRP levels independently of the glucose-lowering in T2DM and nondiabetic patients with increased hs-CRP levels [182, 183]. This suggests that hs-CRP levels in T2DM might not necessarily establish a cause-effect pathophysiological association.

Although the clinical relationship between diabetes and increased level of CRP is well established, the molecular mechanisms by which CRP potentially induces diabetes are yet to be clarified. Particular progress has been made in investigating multiple therapeutic approaches targeting different inflammatory factors [177]. Treatment of T2DM patients with IL-1 receptor blocker [184, 185] or IL-1*β* antibodies [186–189] reduced levels of IL-6 and CRP as markers of systemic inflammation, with simultaneous improvement of glycemia and insulin secretion. Treatment of obese and diabetic subjects with I*κ*B kinase complex *β* (IKK*β*)/nuclear factor *κ*B (NF-*κ*B) inhibitor, as a central proinflammatory player, decreased the level of CRP and improved insulin sensitivity and glycemia [190, 191]. Also, some studies showed beneficial effects of TNF antagonism therapy on CRP level reduction, with a tendency to improve *β* cell function, but without impact on insulin sensitivity [192, 193].

## 5. Conclusions

Numerous prospective studies reported the association between serum CRP level and risk of incident T2DM. However, there is heterogeneity between studies, with some showing an independent positive association of CRP with T2DM incidence [1, 19, 20], while others demonstrate no association after adjustment for adiposity and IR [20, 25]. Since it is unequivocally associated with the development of prediabetes and diabetes-induced vascular complications, the elevated CRP might be an indirect risk factor for T2DM progression. Additionally, elevated CRP levels should be considered one more parameter in the overall assessment of T2DM risk, besides elevated FPG and HbA1C levels, abnormal OGTT, hyperinsulinemia, etc. Thus, elevated CRP levels in patients with higher T2DM risk must warn the clinician to perform available diagnostic procedures to confirm diabetes [194, 195]. Since the incidence of T2DM is expected to increase in the following years dramatically [2], further analysis of the CRP and diabetes association is needed to provide an adjunctive method for early detection of risk for this disease.

## Figures and Tables

**Table 1:** CRP values in animal model studies.

Animal model	T2DM induction	CRP values	Diabetes-associated disorders	Ref.
Sprague-Dawley male rats	High-glucose/high-fat diets (15 weeks), and streptozotocin (single dose 30 mg/kg i.p.)	Increased	Irregular cardiac muscle fibers and degenerative necrosis	[148]
Sprague-Dawley male rats	High-fat diet (10 weeks), and streptozotocin (single dose 25 mg/kg, i.p.)	Increased	Altered oxidative stress parameters in the pancreas and serum	[196]
Sprague-Dawley male rats	High-fat and high-sugar (5 weeks), and streptozotocin (7 days, dose 30 mg/kg, i.p.)	Increased	Injury of the intestinal mucosa reduced antioxidant capacity	[197]
Wistar male rats	High-fat diet (10 weeks), and streptozotocin (single dose 40 mg/kg i.p.)	Increased	Elevated systolic blood pressure, congested blood vessels, necrosis, and inflammation of the heart, pancreas, liver, and kidney.	[198]
Wistar male rats	High-fat diet (10 weeks) and alloxan (single dose 50 mg/kg, i.p.)	Similar to the control values	Physiological pancreas cytoarchitecture, altered lipid profile	[151]
Wistar male rats	Streptozotocin (60 mg/kg i.p.)	Increased	Aorta smooth muscle cell proliferation, altered lipid profile	[21]
Wistar male rats	Streptozotocin (single dose 55 mg/kg i.p.)	Increased	Increased cytokine levels	[22]
Sprague-Dawley male rats	Streptozotocin (two-dose within two days 40 mg/kg i.p.)	Increased	Endothelial dysfunction, modified protein profile	[149]
Wistar male rats	Alloxan monohydrate (single dose 120 mg/kg i.p.)	Increased	Hypercholesterolemia	[150]
Wistar male rats	High-fat, high-fructose, high-casein diet (3 months) and streptozotocin (60 mg/kg in 2 equally doses with a 12 h interval i.p. and 20–30 mg/kg after 2 weeks for some animals)	Increased	Systolic blood pressure deviations	[155]
Wistar male rats	Streptozotocin (single dose 55 mg/kg i.p.)	Increased	Altered lipid profile increased glycosylated hemoglobin and liver enzymes, decreased levels of antioxidant enzymes	[156]
Wistar male rats	Alloxan monohydrate (single dose 120 mg/kg i.p.)	Increased	Altered oxidative stress parameters in serum	[153]
Albino rats	Alloxan monohydrate (single dose 120 mg/kg i.p.)	Increased	Altered oxidative stress parameters in serum, pathological pancreas cytoarchitecture	[154]
Wistar male rats	Streptozotocin three doses (for 3 continuous days 45 mg/kg i.p.)	Increased	Altered oxidative stress parameters in the kidneys	[199]

CRP: C-reactive protein; T2DM: type 2 diabetes mellitus.

**Table 2:** CRP values in human studies.

Subjects	CRP values	Association of CRP and T2DM incidence	Ref
Men	Increased	Positive	[10]
Women	Increased	Positive	[19]
Man and women	Increased	Positive	[9]
Man and women	Non-significant	Positive	[158]
Man and women	Increased	Positive	[163]
Man and women	Increased	Positive	[164]
Man and women	Not measured	Positive	[165]
Men and women	Increased	Not measured	[170]
Men and women	Increased	Non-significant	[1]
Men and women	Increased	Positive	[173]
Men and women	Increased	Positive	[176]

CRP: C-reactive protein; T2DM: type 2 diabetes mellitus.
